# Prevalence and morbidity of neck pain: a cross-sectional study of 3000 elderly men

**DOI:** 10.1186/s13018-023-03508-y

**Published:** 2023-01-13

**Authors:** Henrik Damm, Anette Jönsson, Björn E. Rosengren, Lars Jehpsson, Claes Ohlsson, Eva Ribom, Dan Mellström, Magnus K. Karlsson

**Affiliations:** 1 Clinical and Molecular Osteoporosis Research Unit, Departments of Orthopaedics and Clinical Sciences, Skåne University Hospital, Lund University, 205 02 Malmö, Sweden; 2 Sahlgrenska Osteoporosis Centre, Center for Bone Research, Departments of Internal Medicine and Geriatrics, Sahlgrenska University Hospital, Gothenburg University, Göteborg, Sweden; 3 Section of Orthopaedics, Department of Surgical Sciences, Uppsala University, Uppsala, Sweden; 4 Departments of Internal Medicine and Geriatrics, Sahlgrenska University Hospital, Gothenburg University, Göteborg, Sweden; 5 Department of Drug Treatment, Region Västra Götaland, Sahlgrenska University Hospital, Gothenburg University, Göteborg, Sweden

**Keywords:** Ageing, Elderly, Epidemiology, Men, Motoric symptoms, Musculoskeletal pain, Neck pain, Population-based, Rhizopathy, Thoracolumbar

## Abstract

**Background:**

The purpose of this study is to determine the prevalence and morbidity of neck pain with or without cervical rhizopathy, upper extremity motor deficit and/or thoracolumbar pain in elderly men.

**Methods:**

We conducted a cross-sectional questionnaire study of 3,000 community-dwelling older men with a mean age of 75.4 ± 3.2 years (range 69–81) to determine if they had experienced neck pain with or without cervical rhizopathy/upper extremity motor deficit/thoracolumbar pain (yes/no) during the preceding 12 months, and if so, morbidity with the condition (no/minor/moderate/severe).

**Results:**

Among the participants, 865 (29%) reported they had experienced neck and 1,619 (54%) thoracolumbar pain. Among the men with neck pain, 59% had experienced only neck pain, 17% neck pain and cervical rhizopathy and 24% neck pain, rhizopathy and motor deficit. For men with only neck pain, the morbidity was severe in 13%, for men with neck pain and rhizopathy it was 24%, and for men with pain, rhizopathy and motor deficit it was 46% (*p* < 0.001). Among the men with neck pain, 23% had experienced only neck pain and no thoracolumbar pain; the remaining 77% had both neck and thoracolumbar pain. The morbidity was severe in 10% of the men with neck pain but no thoracolumbar pain and 30% in men with neck and thoracolumbar pain (*p* < 0.001).

**Conclusion:**

Neck pain in elderly men is common but symptoms and morbidity vary. For men who only have neck pain, 1/8 rated their morbidity as severe, while almost half who also had cervical rhizopathy and motor deficit and almost 1/3 of those who also had thoracolumbar pain reported severe morbidity.

## Background

Neck and back pain confer major musculoskeletal health problems worldwide [1–5]. In the USA, lower back pain is the number-one leading cause of spending years living with disability, and neck pain the sixth [6]. Despite the burden, both on the individual and society, the prevalence is debated, and differs among populations and ages [3–5]. In most reports, the 12-month prevalence varies between 17 and 70% [1, 2, 7, 8]. The difference may also depend on different studies using different methods and measurements to identify and define neck pain [2, 7, 8]. However, we found no study that specifically evaluates the one-year prevalence of neck pain with and without cervical rhizopathy/motor deficit in the upper extremity/thoracolumbar pain in elderly men.

The prevalence and severity of lower back pain in elderly men, with and without radiating pain (sciatica), and with or without neurological deficit, has been well identified, with higher morbidity in patients with back pain with rhizopathy/neurological deficit [9]. This is not unexpected, as rhizopathy often confers a sharp radiating pain that follows the distribution of a specific nerve root, in many cases caused by a nerve root compression. If the nerve root compression is accompanied by motor deficit, such as clumsiness and/or weakness, the patient experiences even greater morbidity [9].To our knowledge, similar information regarding neck pain in elderly men is not available and only few publications have examined the association between neck and thoracolumbar pain [10, 11].

Currently, we know that female sex, higher age, social factors, low income, low education, poor psychological health, occupational tasks and impaired quality of life are associated with neck pain [1, 2, 7, 11–18]. As the western world faces a demographic shift, with an increasing older population, we can expect that the prevalence of neck pain will increase [1, 10]. Furthermore, secular changes in social, educational, psychological and occupational lifestyle factors imply that the prevalence of neck pain may have changed and needs to be re-examined. Current and up-to-date information may aid health care planning as well as facilitate identification of patient groups with high morbidity in need of targeted preventive or curative interventions.

We hypothesised that neck pain in elderly men is a common condition, and thus a problem of magnitude even if the pain may be less severe. We also hypothesised that if the neck pain is accompanied by cervical rhizopathy and/or motor deficit and/or thoracolumbar pain, the morbidity will be more severe. With this background, our rationale of the study was to (i) identify in elderly men the 12-month prevalence of neck pain, with and without cervical rhizopathy/motor deficit/thoracolumbar pain. That is, all men had neck pain but only some additional cervical rhizopathy and/or motor deficit and/or thoracolumbar pain, (ii) identify the morbidity in the subgroups.

## Methods

We collected the data for this study from The Osteoporotic Fracture in Men (MrOs) Study Sweden. This is a multicentre study that includes 3,014 men aged 69–81, recruited at medical centres in Gothenburg (*n* = 1010), Malmö (*n* = 1005) and Uppsala (*n* = 999). The primary aim of the project is to specifically in men identify risk factors for osteoporosis, falls and fractures, in a prospective observational study design, explaining why no women were evaluated in this study. The study design has been described in detail in previous publications [19–21]. In summary, the men invited to participate in MrOs Sweden were randomly chosen from the national population register. Since MrOs Sweden is a sub-cohort of MrOS International, and since the study is observational with a variety of endpoint variables, no specific power calculations were done before study start. The men were invited to the study through surface mail, with a letter where they were asked to respond, whether or not they wanted to participate. Individuals who did not respond were sent a second letter. If there was no response to the second letter, we tried to reach them through a telephone call. Those who did not answer any letter or telephone call were classified as non-responders. All who wanted to participate were then scheduled for a baseline evaluation in our research laboratories, the evaluation used in this study. The attendance rate was 45%, with all baseline exams conducted from 2001 to 2004. To be eligible for the study, the men had to be community living in the vicinity of the medical centres, able to walk without aids, not have bilateral hip prostheses, be able to provide self-reported data and be able to give signed informed consent. No other exclusion criteria were used. These inclusion criteria were defined because the primary aim of the MrOS project was to evaluate risk factors, including previous lifestyle, for osteoporosis and fractures, especially hip fractures, in community-living men.

We used cross-sectional data from the baseline exam in this study. Height and weight were measured using standard equipment. We performed two consecutive measurements of height in the same session and used the average. In case of a discrepancy of ≥ 5 mm between the measurements, we performed a third measurement, and used the average of the two nearest values. Body mass index (BMI) was calculated as the weight divided by the square of the height (kg/m^2^).

**Fig. 1:**
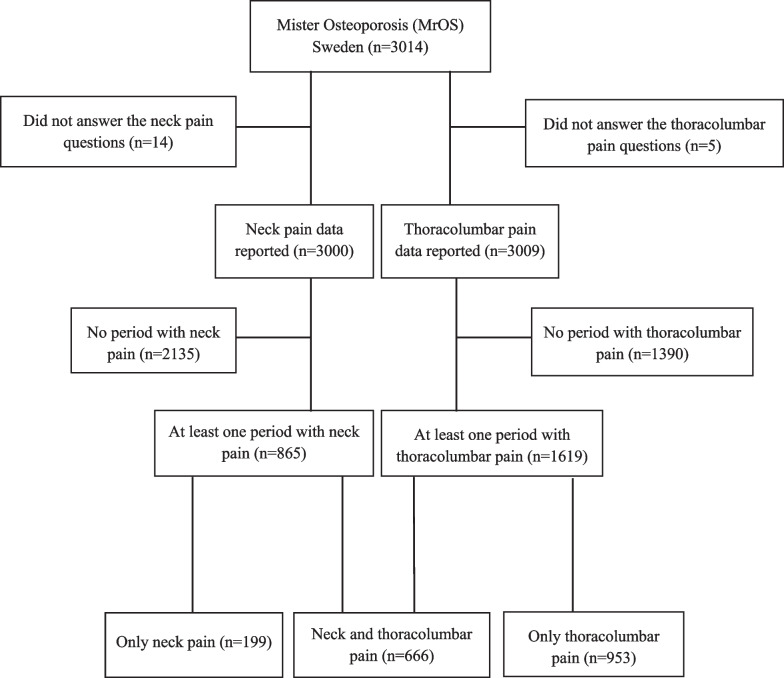
Flow chart of study participants

At the baseline exam, all participants were also asked to answer a questionnaire on lifestyle. These questions (the outcome measures) included questions regarding neck and thoracolumbar pain [19–21]. Fourteen men did not answer these questions (Fig. 1). There were no clinical examinations, nor objective measurements of sensory or motor deterioration in the patients.

The pain questions assessed whether the men had experienced neck pain (defined as any type of pain in the neck region), cervical rhizopathy (defined as any type of radiating pain and/or tingling in arm/hand), upper extremity motor deficit (defined as any type of subjective weakness or paresis in the upper extremity) and thoracolumbar pain (defined as any type of pain in the thoracic and/or lumbar regions, yes/no) during the 12 months that preceded the baseline exam. Thus, in this study design it was not possible to state any causality of the symptoms. Those who answered yes to any question were further asked to grade the severity of the symptom (no, mild, moderate or severe pain). That is, the grading of severity was entirely based on the subjective estimation of the morbidity. All forwarded questions are shown in Appendix 1. Since the outcome measures only include the participants’ own subjective estimates, we only report their answers with no further interpretation. That is, the outcome measured in this study reflects the patient-reported subjective estimation of pain and morbidity.

We presented data after conducting three stratifications. In the first, we categorised the men in four age classes (i) 69–72 years, (ii) 73–75 years, (iii) 76–78 years and (iv) 79–81 years. In the second, we categorised the men in three groups depending on a history of neck pain/rhizopathy/motor deficit in (i) men with only neck pain but no rhizopathy/motor deficit, (ii) men with neck pain and rhizopathy but no motor deficit and (iii) men with neck pain, rhizopathy and motor deficit (Appendix 2) (Fig. 1). In the third, we categorised the men in two groups depending on a history of neck/thoracolumbar pain in (i) men with neck pain (with or without rhizopathy/motor deficit) but no thoracolumbar pain and (ii) men with neck (with or without rhizopathy/motor deficit) and thoracolumbar pain (Appendix 2) (Fig. 1).

We used Statistical Package of Social Science (SPSS version 25, IBM, Armonk, NY, USA) for statistical analysis. Categorical variables are presented as numbers (*n*) and proportions (%) and continuous variables as means ± standard deviations (SD). We used the Chi-square test, to examine group differences and regarded *p* < 0.05 as a statistically significant difference. The Ethics Committees and the radiological committee at each centre approved the study (LU 693-00, Gbg 014-01, Ups 01-057). All participants gave written informed consent before study start and the study was performed in accordance with the Declaration of Helsinki.

## Results

### Epidemiology

Table 1 shows the background data of men with and without neck pain: 865 (29%) of the men had experienced at least one episode of neck pain during the 12-month period and 1,619 (54%) had at least one episode of thoracolumbar pain (Fig. 1). We found similar proportions of men with neck pain in the different age groups (Table 2).

**Table 1:** Baseline characteristics in 3000 elderly men aged 69–81 in relation to if they had neck pain or not during the preceding 12-month period

	Men with neck pain (865)	Men with no neck pain (2135)
**Anthropometry**		
Age (years)	75.4 ± 3.2 (865)	75.5 ± 3.2 (2135)
Height (centimetre)	174.9 ± 6.5 (865)	174.7 ± 6.6 (2135)
Weight (kg)	81.2 ± 12.0 (865)	80.6 ± 12.1 (2135)
BMI (kg/m^2^)	26.5 ± 3.6 (865)	26.4 ± 3.5 (2135)
**Lifestyle factors**		
Daily exercise walking distance (km/day)	3.7 ± 2.2 (559)	4.0 ± 2.4 (1381)
Smoking	10.2% (590)	13.9% (1378)
Alcohol:		
0 drinks/week	20.3% (171)	20.5% (430)
> 0 to < 2 drinks/week	27.0% (228)	22.5% (471)
2 or more drinks/week	52.7% (445)	57.0% (1193)
**Associated factors**		
Diabetes	10.6% (865)	9.0% (2133)
Osteoporosis	2.3% (865)	17% (2129)
Stroke	7.1% (863)	6.2% (2128)
Parkinson’s disease	1.0% (862)	0.7% (2121)
Cancer	17.5% (864)	14.7% (2133)
Gout, joint inflammation or arthritis	25.5% (850)	16.0% (2117)
Chronic bronchitis, asthma or emphysema	10.2% (861)	7.7% (2126)
High blood pressure	37.6% (860)	35.6% (2126)
Myocardial infarction	15.1% (857)	13.9% (2129)
Angina	19.5% (860)	13.3% (2122)
Congestive heart failure	10.9% (857)	9.1% (2114)
Fallen during the previous 12 months	18.7% (861)	15.6% (2130)
Fracture during the previous 12 months	1.5% (852)	1.2% (2110)
Born outside Sweden	8.4% (864)	6.5% (2134)
Education:		
Public school (7 years)	47.7% (411)	45.8% (976)
Primary school or equivalent (9 years)	12.6% (109)	11.2% (240)
High school	19.5% (168)	20.0% (425)
University/college	20.2% (174)	23.0% (489)

Data are provided as means ± standard deviations (SD) or as proportions (%) with number of data/answers (*n*) for each variable within brackets

**Table 2:** Prevalence of neck pain in 3000 men during the preceding 12 months in relation to age

	Age 69–72 (800)	Age 73–75 (985)	Age 76–78 (687)	Age 79–81 (528)	*p*-value
Neck pain (865)	30.3% (242)	29.0% (286)	27.9% (192)	27.5% (145)	0.67
No neck pain (2135)	69.8% (558)	71.0% (699)	72.1% (495)	72.5% (383)	

Data are presented as proportion (%) with numbers (*n*) within brackets

Among the 865 men with neck pain, 507 (59%) had experienced only neck pain, 148 (17%) neck pain with rhizopathy but no motor deficit and 210 (24%) neck pain with rhizopathy and motor deficit. We found similar proportions of men with neck pain/cervical rhizopathy/upper extremity motor deficit in the different age groups (Table 3).

**Table 3:** Prevalence of men with only neck pain, neck pain and cervical rhizopathy or neck pain, rhizopathy and motor deficit and prevalence of men with neck pain but no thoracolumbar pain or neck pain and thoracolumbar pain among the 865 men with neck pain during the preceding 12-month period in relation to age

	Age 69–72 (242)	Age 73–75 (286)	Age 76–78 (192)	Age 79–81 (145)	*p*-value
**Neck pain/cervical rhizopathy/motor deficit**					
Neck pain with no rhizopathy/motor deficit (507)	56.6% (137)	63.3% (181)	57.8% (111)	53.8% (78)	0.13
Neck pain with rhizopathy/no motor deficit (148)	19.4% (47)	15.4% (44)	13.0% (25)	22.1% (32)	
Neck pain with rhizopathy and motor deficit (210)	24.0% (58)	21.3% (61)	29.2% (56)	24.1% (35)	
**Neck pain/thoracolumbar pain**					
Neck pain but no thoracolumbar pain (199)	21.1% (51)	26.9% (77)	22.9% (44)	18.6% (27)	0.21
Neck pain and thoracolumbar pain (666)	78.9% (191)	73.1% (209)	77.1% (148)	81.4% (118)	

Data are presented as proportions (%) with numbers (*n*) within brackets

Among the 865 men with neck pain, 199 (23%) had experienced neck pain but no thoracolumbar pain and 666 (77%) neck and thoracolumbar pain. We found similar proportions of men with regional pain distribution in the different age groups (Table 3).

### Morbidity, neck pain, rhizopathy and/or motor deficit

Among the 864 men with neck pain (with or without rhizopathy and/or motor deficit) and with the morbidity scored, 313 (36%) reported no or minor morbidity, 352 (41%) moderate and 197 (23%) severe morbidity. We found similar proportions of men with no or minor/moderate/severe morbidity in the different age groups (Table 4).

**Table 4:** Prevalence of men with no/minor, moderate and severe morbidity among the 864 men (data of severity missing in 1/865 men) who graded neck pain severity (with and without rhizopathy and/or motor deficit) during the preceding 12-month period in relation to age

	Age 69–72 (242)	Age 73–75 (285)	Age 76–78 (192)	Age 79–81 (145)	*p*-value
No/minor morbidity (313)	35.5% (86)	35.8% (102)	37.0% (71)	37.2% (54)	0.99
Moderate morbidity (354)	41.3% (100)	42.8% (122)	39.1% (75)	39.3% (57)	
Severe morbidity (197)	23.1% (56)	21.4% (61)	24.0% (46)	23.4% (34)	

Data are presented as proportions (%) with numbers (*n*) within brackets

Among the 506 men with only neck pain and with the morbidity scored, 65 (13%) graded the morbidity as severe, while 35 (24%) among the 148 men with neck pain and rhizopathy but no motor deficit. Among the 209 men with neck pain and rhizopathy and motor deficit, 97 (46%) graded the morbidity as severe (*p* < 0.001) (Table 5).

**Table 5:** Prevalence of men with no/minor, moderate or severe morbidity in the 864 men (data of severity missing in 1/865 men) with neck pain severity graded during the preceding 12 months categorised in those with only neck pain, neck pain and cervical rhizopathy or neck pain with cervical rhizopathy and motor deficit

	No/minor morbidity (313)	Moderate morbidity (354)	Severe morbidity (197)	*p*-value
Neck pain with no rhizopathy/motor deficit (506)	46.4% (235)	40.7% (206)	12.8% (65)	< 0.001
Neck pain with rhizopathy/no motor deficit (148)	31.8% (47)	44.6% (66)	23.6% (35)	
Neck pain with rhizopathy and motor deficit (209)	14.8% (31)	39.0% (82)	46.2% (97)	

Data are presented as proportion (%) with numbers (*n*) within brackets

### Morbidity, neck pain, thoracolumbar pain

Among the 198 men with neck pain (with or without rhizopathy and/or motor deficit) but no thoracolumbar pain and with the morbidity scored, 104 (53%) reported the morbidity to be none or minor, 74 (37%) moderate and 20 (10%) severe, compared to 118 (18%), 350 (52%) and 198 (30%), respectively, in the 666 men with neck (with or without rhizopathy and/or motor deficit) and thoracolumbar pain and with the morbidity scored (*p* < 0.001) (Table 6).

**Table 6:** Prevalence of men with no/minor, moderate or severe morbidity in the 864 men (data of severity missing in 1/865 men) who graded their neck pain severity during the preceding 12 months categorised in those with neck pain (with and without rhizopathy and/or motor deficit) but no thoracolumbar pain and in those with neck pain and thoracolumbar pain

	No/minor morbidity (222)	Moderate morbidity (424)	Severe morbidity (218)	*p*-value
Neck pain with no thoracolumbar pain (198)	52.5% (104)	37.4% (74)	10.1% (20)	< 0.001
Neck pain with additional thoracolumbar pain (666)	17.7% (118)	52.6% (350)	29.7% (198)	

Data are presented as proportion (%) with numbers (*n*) within brackets

Among the 198 men who had experienced both neck and thoracolumbar pain and had rated the morbidity as severe, 78 (39%) had severe neck pain but no/minor or moderate thoracolumbar pain, 53 (27%) had both severe neck and severe thoracolumbar pain, and 67 (34%) had severe thoracolumbar pain but no/minor or moderate neck pain (*p* < 0,001).

## Discussion

In this study, we found that close to one-third of community-living elderly men experienced neck pain during a 12-month period, but that more than 75% rated the morbidity to be none, minor or moderate. We also found that the proportion of men who rated the morbidity as severe was higher if the neck pain was accompanied by rhizopathy, motor deficit or thoracolumbar pain.

The one-year prevalence of neck pain in the adult population varied greatly between different studies, with some studies reporting prevalence as low as 16% and others as high as 75% [1, 8, 10, 13]. However, the lack of a general definition of neck pain makes the utility of results and the comparison between studies difficult. There is a need for epidemiological studies in many populations, including in different settings and age groups. Nonetheless, our 12-month neck pain prevalence of 29% was similar to the 12-month neck pain prevalence in a Greek urban population of men and women aged 15–65 years [22] and a British population of men and women aged 16–64 years [23] (one-year neck pain prevalence 29% and 34% respectively). It was also similar to a Russian rural and urban population of men and women aged 40–94 years (mean lifetime neck pain prevalence 29%) [24]. Previously reported 12-month prevalence from different Swedish populations vary; Bergman et al. found a one-year neck pain prevalence of 14.5% in men between 20 and 74 years of age [25], while Linton et al. reported a much higher one-year prevalence, 66.3% in men and women 35–45 years of age [26]. The latter study made no clear distinction between back or neck pain, which may explain the high prevalence. To facilitate comparisons between studies of neck pain, the anatomic region of the neck needs to be defined in the same way, for example as defined by Guzman et al. [27].

Reports from other populations found a lower prevalence. For example, Genebra et al. reported one-year neck pain prevalence to be 20% in a Brazilian population of men and women older than 20 years [13] and Palacios-Ceña et al. reported a one-year neck pain prevalence of 16% in a Spanish population of men and women older than 16 years [10]. Since the prevalence of musculoskeletal disorders, including neck pain, differs and mainly increases with age [5, 10, 13], all comparisons between studies must take the age distribution of participants into account. According to a systematic analysis of the Global Burden of Disease Study, the point prevalence of neck pain increases with age until a cut-off of 70–74 years, where the prevalence decreases [3]. This, together with the narrow age span in our study compared with wider age spans in the above-mentioned studies, might explain why we did not find any influence of age on prevalence or severity of neck pain. This is in contrast to other published studies [1, 2, 7, 11, 13, 16–18]. Differences in the age of participants may thus have contributed to the higher prevalence in our study compared to those from Brazil and Spain.

Our study only includes elderly men, while the above-mentioned studies also include women; this will also contribute to prevalence differences compared to our study. The female gender has been reported as a risk factor for neck pain in various studies, but more recent epidemiological studies question whether this is true [5, 28]. We could not find any previous study of neck pain that focuses on elderly men > 69 years. As neck pain is a multifactorial disease, various risk factors and triggers must be evaluated to better understand how to prevent, diagnose and manage patients with neck pain.

We found that our hypothesis that neck pain accompanied by symptoms of rhizopathy/motor deficit/thoracolumbar pain is associated with a higher morbidity than neck pain alone seems to be true. We were unable to find prevalence and morbidity data in these groups of patients with neck pain in other studies and consider this information clinically important. For example, our results indicate that three times as many men with both neck and thoracolumbar pain rate their morbidity as severe compared to men with neck pain without thoracolumbar pain, and detailed questions on pain localisation may be more essential than previously known in patients with neck pain. It is also of great interest to find that, among men with both neck and thoracolumbar pain who rated the morbidity as severe, 73% rated the pain severity differently in the two anatomic regions. This may also be of clinical importance when identifying subgroups in need of specific targeted preventive and curative interventions. This also highlights the necessity of categorising patients with neck pain in future studies, i.e. with only neck pain or also with accompanying symptoms such as rhizopathy/motor deficit/thoracolumbar pain, to make comparisons of the prevalence and morbidity data across studies meaningful.

The strengths of this study include the large population-based sample with a high participation rate of elderly community-living men within a narrow age span. Therefore, we are of the opinion that the data can be generalised to similar cohorts, However, we must also emphasise that our inferences cannot be directly transferred to cohorts of younger or older men, men of other ethnic backgrounds, men living in other societies or women.

Study weaknesses include the inclusion of only ambulatory elderly men without bilateral hip replacements. Further, despite the population-based study design, we cannot exclude selection bias. We speculate that unhealthy and sick men may have declined participation, but we have no indication that this proportion is different in men with and without neck pain. In addition, the majority of our cohort were of Caucasian ethnicity within a small age span, and all lived in Sweden. Therefore, we cannot state that our inferences account for men of other ethnicities, men living in other countries and in men of other ages. Furthermore, with our approach, temporal and causal relationships are not possible to establish. We can only draw conclusions regarding associations. The limitations also include the retrospective study design that yields a risk of recall bias and the subjective patient-reported morbidity. It is also possible that the sensory symptoms occur due to nerve entrapment in the arms and not the neck, and the motoric symptoms are due to other disorders than nerve root compression in the neck. As the study was done without clinical examinations or other diagnostic tools, we can only report the subjective estimates by the patients. It would thus have been an advantage to have prospectively collected information on neck pain and limitations of daily living (ADL), instead of relying on subjective reported data. It would also have been advantageous to evaluate the number and duration of each neck pain episode, frequency of associated symptoms and recurrence rate.

## Conclusions

Neck pain in elderly men is common but symptoms and morbidity vary. For men who only have neck pain, 1/8 rate their morbidity as severe while almost half of those who also have rhizopathy and upper extremity motor deficit report severe morbidity. One-third of those with neck pain and thoracolumbar pain report severe morbidity. Future studies on neck pain should examine risk factors for high morbidity and impact on ADL not only in men but also in women and in various age groups and societies. Preferably, studies should use the categories employed in this study, i.e. with/without rhizopathy, with/without motor deficit and with/without thoracolumbar pain, to identify groups suitable for preventive and curative interventions.

## Data Availability

The data that support the findings of this study are available on request from the corresponding author H.D. The data are not publicly available because their contents could compromise research participant privacy/consent.

## Appendix 1: The questions addressing neck pain and associated morbidity

Have you during the last 12 months:

*Question 1*: a. had neck pain? (yes/no). b. If yes, how troublesome? (none/minor/moderate/sever).
*Question 2*: *a.* had neck pain with pain radiation in arm and/or hand? (yes/no). b. If yes, how troublesome? (none/minor/moderate/sever).
*Question 3*: a. felt numbness and tingling in arm and hand? (yes/no). b. If yes, how troublesome? (none/minor/moderate/sever).
*Question 4*: *a.* felt weakness in arm and hand? (yes/no). b. If yes, how troublesome? (none/minor/moderate/sever).
*Question 5*: *a.* felt clumsiness when you use your hands? (yes/no). b. If yes, how troublesome? (none/minor/moderate/sever).
*Question 6*: *a.* had back pain? (yes/no). b. If yes, how troublesome? (none/minor/moderate/sever).
*Question 7*: *a.* had lower back pain? (yes/no). b. If yes, how troublesome? (none/minor/moderate/sever).

## Appendix 2: Definition of the subgroups in relation to the questions

Neck pain/cervical rhizopathy/motor disturbances in upper extremity.

- Localised neck pain: *Question 1—*Yes; *Question 2—*No; *Question 3—*No.
- Neck pain/cervical rhizopathy: *Question 1—*Yes; *Question 2 and/or Question 3—*Yes
- Neck pain/cervical rhizopathy/motor disturbances: *Question 1—*Yes; *Question 2* and/or *Question 3—*Yes and *Question 4 and/or Question 5—*Yes

Neck and thoracolumbar pain.

- Neck pain but no thoracolumbar pain: *Question 1—*Yes; *Question 6—*No; *Question 7—*No
- Neck pain and thoracolumbar pain: *Question 1—*Yes; *Question 6 and/or Question 7—*Yes
